# The relationship between social deprivation and a frailty index of cumulative deficits in French middle-aged caregivers

**DOI:** 10.1186/s12877-021-02736-3

**Published:** 2022-01-03

**Authors:** Jonathan Giovannelli, Anthony Pinon, Manon Lenain, Anne-Laure Cleys, Brigitte Lefebvre, Nicolas Capon, Simon Spychala, Eric Boulanger, Pascaline Cassagnaud, Mael Barthoulot

**Affiliations:** 1 GIOVANNELLI Epidemiology and Clinical Research Counselling, 93 rue du 20ème siècle, 59160 Lille, France; 2 Institut Pasteur de Lille, Lille, France; 3 CHU de Lille, Lille, France

**Keywords:** Frailty, Deficits, Caregiver, Middle age, Social deprivation, Epices score

## Abstract

**Background:**

The Institut Pasteur de Lille, in the north of France, has implemented a large, multidisciplinary health check, which aims to identify frailty in middle-aged caregivers. We aimed to construct an adapted frailty index of cumulative deficit (FI-CD) and study the associated factors, in particular socioeconomic factors.

**Methods:**

The cross-sectional study included caregivers aged 45 to 65. A 34-item FI-CD including deficits adapted to a middle-aged population (related to cognition and autonomy, dietetics, physical activity, comorbidities, functional signs, lab values and paraclinical examinations) was constructed in accordance with standard procedures. It was calculated as a ratio of deficits present out of the total number of possible deficits, giving a continuous score between 0 and 1. Scores > 0.25 and >  0.4 were classified as frailty and severe frailty, respectively. Univariate and multivariate associations were studied using linear regressions.

**Results:**

One hundred and seventeen caregivers were included; among them, 111 were analyzed due to missing values. The mean FI-CD was 0.22 ± 0.08. Forty (36%) individuals were classified as frailty and three (2.7%) as severe frailty. In multivariate analysis, FI-CD was significantly associated with age (beta [95% confidence interval] = 0.005 [0.002; 0.009] per 1-year increase, *p* = 0.005) and social deprivation (beta = 0.054 [0.007; 0.102], *p* = 0.025). A significant interaction was observed between and age and social deprivation (*p* = 0.036). The adjusted relationship between FI-CD and age was beta = 0.010 [0.002; 0.019], *p* = 0.017 in precarious caregivers, and beta = 0.003 [− 0.001; 0.007], *p* = 0.19 in non-precarious caregivers.

**Conclusions:**

The study suggested that the 34-item FI-CD could have clinical utility in the management of middle-aged caregivers. Social deprivation appeared as an important factor associated with frailty, highlighting the importance of early care and social support for precarious caregivers.

**Supplementary Information:**

The online version contains supplementary material available at 10.1186/s12877-021-02736-3.

## Background

Frailty is characterized by a decline in functioning across multiple physiological systems, accompanied by an increased vulnerability to stressors [1]. Because of the ageing population, it becomes a global health burden, with major implications for clinical practice and public health, with a prevalence expected to rise rapidly. All people are at risk of developing frailty, with a higher risk in individuals with comorbidities, low socioeconomic condition, poor diet, and sedentary lifestyles [2].

The Institut Pasteur of Lille, in the north of France, has implemented a prevention program, which aims to identify and correct pre-frailty or frailty in middle-aged individuals, to age better and limit the consequences of ageing. The first step of this prevention program is a large, multidisciplinary health check, which aims to identify frailty. It includes a medical check with numerous clinical and paraclinical examinations (bone mineral density, dual energy X-ray absorptiometry, spirometry, visual and auditory examinations, electrocardiogram, biology), and interviews carried out by neuropsychologists, dieticians, and medico-sports educators. The second step is a coaching intervention, which aims to try to correct the observed frailty.

This program is particularly aimed at caregivers. Indeed, numerous studies have shown that caregiving can adversely influence the caregiver’s psychological and physical health. More than the general population, caregivers may suffer from stress and depression and are less likely to engage in preventive health behaviors, which may increase the risk for mortality [3–6]. Furthermore, French caregiver assistance policy comes late compared to the progress made in other countries, in particular Northern, European countries. Today, there is still little individualized support for caregivers in France [7]. Data from the Institut Pasteur of Lille make it possible to describe this particular population and to develop a useful tool to assess its frailty.

To help identify frailty individuals in this middle-aged population, the implementation of an adapted frailty score would be useful. Today, several tools exist to try to measure frailty (e.g. the wildly used clinical frailty score described by Fried et al. [1] or the Groningen Activity Restriction Scale [8]), without consensus to define the best one [9]. A solution that seems to be adapted to our large, multidisciplinary health check is the construction of a frailty index of cumulative deficits (FI-CD). The FI-CD was firstly designed by Rockwood et al. [10, 11]. It involves the accumulation of 30 or more comorbidities, symptoms, diseases, disabilities or any deficiency in health with the idea that a greater number of health deficits indicates higher frailty [12]. It is well validated, and has been applied to multiple datasets [9]. In addition, the frailty index would allow the study of factors associated with frailty, in particular socioeconomic factors, in order to identify sub-populations at risk and guide the future recruitment of caregivers to offer the Institut Pasteur de Lille’s prevention program to the most vulnerable populations, who have a high probability of suffering the consequences of aging.

Thus, we aimed to construct an adapted FI-CD, in French middle-aged caregivers participating to the health check of the Institut Pasteur of Lille, and study the associated factors, in particular socioeconomic factors.

## Methods

### Population and study design

This is a cross-sectional study including all caregivers aged 45 to 65, who participated in the health check of the Institut Pasteur of Lille, from April 2018 to April 2020. There were no exclusion criteria. All individuals who participated in this health check were volunteers and were recruited from different sources, including the respite platform for caregivers (“Maison des aidants”), the neurology and geriatric departments of the University hospital of Lille, as well as caregivers who learned about this program through in the media and who had been invited to contact the Institut Pasteur of Lille to participate in a prevention program.

The study was conducted in accordance with the Declaration of Helsinki and the French law relative to clinical non-interventional research. Consent was obtained from each caregiver included in the study for the use of de-identified medical data. Moreover, data use and confidentiality was ensured in accordance with reference method MR-004 of the French commission for data protection (Commission Nationale Informatique et Liberté, number 2220128 v 0).

### Construction of the frailty index cumulative deficits

A standard procedure for the construction of the FI-CD was proposed by Searle et al. [12]. The FI-CD is calculated as a ratio of deficits present out of the total number of possible deficits, giving a continuous score between 0 and 1. Deficits are variables that must include a broad range of systems. In brief, all variables must be health-related and age-associated, neither overly common, nor overly uncommon (< 80% in this study). All variables included in the frailty index were recoded such that 0 signified the absence of a deficit, while the presence of the deficit was given a score of 1. An intermediate deficit was coded 0.5 (e.g. for the body mass index (BMI): overweight was coded 0.5, while obesity and leanness were coded 1). Any individual who was missing 20% or more of the variables was excluded from the study [13].

Here, we constructed a 34-item FI-CD including deficits adapted to a middle-aged population. They are related to cognition and autonomy, dietetics, physical activity, comorbidities, functional signs, lab values and paraclinical examinations (including bone mineral density, dual energy X-ray absorptiometry, electrocardiogram and pure tone audiometry) (Table 1).

**Table 1:** Variables and cut-points for the Frailty Index

Variables	Coding
**Cognition and autonomy**	
Cognitive assessment (MoCA score)	Cut-points according to age and socio-cultural level (GRECOGVASC): Preservation = 0, Fragility = 1 (MoCA score < − 1 SD) [14, 15]
Processing speed assessment (Coding score)	Cut-points according to age (WAIS-IV): Preservation = 0, Fragility = 1 (Coding score < − 1 SD) [16, 17]
Autonomy assessment (4-item IADL score)	< 1 = 0, ≥ 1 = 1 [18]
Able to manage his/her daily life if alone for 15 days	Yes = 0, No = 1
**Dietetics**	
Overall nutritional intakes	Sufficient = 0, Insufficient = 1
Calcium intakes	Sufficient = 0, Insufficient = 1
Protein intakes	Sufficient = 0, Insufficient = 1
**Physical activity and risk of falling**	
Marshall physical activity assessment score	≥ 4 = 0, < 4 = 1 [19]
One or more falls in the past year	No = 0, Yes = 1
Gait speed test (4-m)	≥ 1 m/s = 0, < 1 m/s = 1 [20]
Handgrip strength test	Men: ≥ 30 kg = 0, < 30 kg = 1; Women: ≥ 20 kg = 0, < 20 kg = 1 [21]
**Comorbidities**	
Cardiovascular disease	No = 0, Yes = 1 (History of myocardial infarction, stroke, heart failure, angina or arteritis of the lower limbs)
Other heart disease	No = 0, Yes = 1 (History of arrhythmia or valvular heart disease)
High blood pressure	No = 0, Yes = 1 (SBP > 140 mmHg or DBP > 90 mmHg or history of HBP)
Diabetes	No = 0, Yes = 1 (HbA1c > 6.5% or history of diabetes)
Dyslipidaemia	No = 0, Yes = 1 (Total cholesterol ≥2 g/l or triglycerides ≥1.5 g/l or history of dyslipidaemia)
Cancer	No = 0, Yes = 1
Airway obstruction	No = 0, Yes = 1 (FEV1/FVC < 0.7 or history of COPD) [22]
Thyroid disease	No = 0, Yes = 1 (Abnormal hs-TSH according to laboratory standards or history of thyroid disease)
Vision disease	No = 0, Yes = 1 (History of cataract, glaucoma or age-related macular degeneration)
Anxiety	HAD < 8 = 0, HAD ≥8 and < 11 = 0.5, HAD ≥11 = 1 [23]
Depression	HAD < 8 = 0, HAD ≥8 and < 11 = 0.5, HAD ≥11 = 1 [23]
**Regular treatment**	No = 0, Yes = 1
**Functional signs**	
Chronic sleep disorders	No = 0, Yes = 1 (Any disorder during at least 3 nights per week, for at least 3 months, despite habits and conditions suitable for sleep)
Difficulty retaining urine	No = 0, Yes = 1
**Lab values and paraclinical examinations**	
Anaemia	No = 0, Yes = 1 (Hb < 13.5 g/100 ml in men, Hb < 12.5 g/100 ml in women)
Elevated hs-CRP	No = 0, Yes = 1 (> 3 mg/l) [24]
Elevated liver enzymes (ALAT, ASAT or GGT)	No = 0, Yes = 1 (According to laboratory standards)
Body mass index	BMI ≥ 18.5 kg/m^2^ and < 25 kg/m^2^ = 0, BMI ≥ 25 kg/m^2^ and < 30 kg/m^2^ = 0.5, BMI < 18.5 kg/m^2^ or ≥ 30 kg/m^2^ = 1 [25]
Pure tone audiometry	PTA4 ≤ 25 dB in each ear = 0, PTA4 > 25 dB in at least one ear = 1 [26]
Hip bone mineral density	Normal (T-score > − 1) = 0, Osteopenia (T-score = [− 1, − 2.5[) = 0.5, Osteoporosis (T-score ≤ − 2.5) = 1 [27]
Lumbar spine bone mineral density	Normal (T-score > − 1) = 0, Osteopenia (T-score = [− 1, − 2.5[) = 0.5, Osteoporosis (T-score ≤ − 2.5) = 1 [27]
Dual energy X-ray absorptiometry	Normal = 0, Sarcopenia = 1 (aLM/Ht^2^ ≤ 7.23 kg/m^2^ in men, ≤ 5.67 kg/m^2^ in women) (20)
Electrocardiogram	No or minor anomalies = 0, Anomalies = 1

*MoCA* Montreal Cognitive Assessment, *GRECOGVASC* Groupe de Réflexion pour l’Évaluation COGnitive Vasculaire, *SD* Standard deviation, *WAIS-IV* Wechsler Adult Intelligence Scale-4th edition, *IADL* Instrumental Activities of Daily Living, *HBP* High Blood Pressure, *SBP* Systolic Blood Pressure, *DBP* Diastolic Blood Pressure, *HbA1c* Haemoglobin a1c Protein, *FEV1* Forced Expiratory Volume in 1 s, *FVC* Forced Vital Capacity, *hs-TSH* high-sensitivity Thyroid Stimulating Hormone, *HAD* Hospital Anxiety and Depression Scale, *Hb* Haemoglobin, *hs-CRP* high-sensitivity C-Reactive Protein, *ASAT* Aspartame Aminotransferase, *ALAT* Alanine Aminotransferase, *GGT* Gamma-GT, *BMI* Body Mass Index, *PTA4* Pure-Tone Average of 0.5, 1.0, 2.0, 4.0 kHz, *dB* Decibel, *aLM/Ht*^*2*^ appendicular fat lean mass/ ht^2^

All clinical and paraclinical examinations took place on the day of the health check-up at the Institut Pasteur de Lille by trained caregivers (physicians, neuropsychologists, dieticians, and medico-sports educators). The variables used for the construction of the FI-CD were retrieved from computerized patient medical records.

For description and sensitivity analysis in the study of associated factors, the FI-CD was also categorized, based on proposed cut-offs: scores > 0.25 and >  0.4 were classified as frailty and severe frailty, respectively [28–30].

### Other measurements

Sociodemographic characteristics, caregiving characteristics and other health characteristics were also obtained from medical records. Social deprivation was evaluated using the Epices score [31], including questions about finance difficulties for basic needs, to be homeowner, marital status, social relations and leisure. A score > 30 defines “precarious individuals”. Moreover, educational level (primary, secondary or tertiary), professional situation (active, inactive or retired) and socio-professional category were recorded. Caregiving characteristics included the relationship to care recipient and the Caregiver Reaction Assessment (CRA) questionnaire [32, 33]. This questionnaire explores five dimensions of caregivers’ reactions: caregiver’s self-esteem problems, lack of family support, financial problems, disrupted schedule and health problems. The CRA questionnaire was implemented during the study, and data were therefore only available for a subsample of caregivers. Health characteristics (outside the FI-CD) included a perceived health Visual Analog Scale (VAS) (0 the worst and 100 the best), financial assistance for long-term illness (a French social assistance), smoking status, Alcohol Use Disorders Identification Test (Audit) questionnaire for alcohol misuse [34], and data on health prevention (general practitioner consulted within the year, screening with mammography, cervical smear and colorectal cancer). The questionnaires were mostly filled out by the participants online, few days before the health check-up; some participants who did not have computer equipment filled it out on the same day of the health check-up.

### Statistical analyses

Compared with previously published indexes, the FI-CD index should have several characteristics: (i) a skewed density distribution, (ii) an accumulation of deficits with age (prior estimate is a rate of about 3% per year), (iii) the presence of a sub-maximal, age-invariant limit (prior estimate is about 0.67), and (iv) an association with mortality [12]. In this study, the number of individuals was too small to calculate a valid age-invariant limit and data on mortality were not available. Firstly, we studied the distribution of the FI-CD using a histogram with a density curve. Secondly, we plotted FI-CD versus age, and graphically evaluated the linearity of the relationship comparing the linear regression line and the locally weighted scatterplot smoothing (Lowess) regression curve. The rate of accumulation of deficits was calculated by evaluating the slope of a best fit log of the FI-CD in relation to age. Thirdly, we studied the association between the FI-CD and other health outcomes (perceived health VAS and financial assistance for long-term illness).

Characteristics of the population were described using mean ± standard deviation (SD), or median [interquartile range (IQR)] in case of non-normality, for quantitative variables, and number (percentage) for qualitative variables. Characteristics of precarious and non-precarious caregivers were compared using t tests, or Wilcoxon’s rank sum tests in case of non-normality, for quantitative variables, and Fisher’s exact tests for qualitative variables.

To study the associations between the FI-CD as the dependent variable and the characteristics of patients as the explanatory variables, we firstly used univariate linear regressions. Then, we built, as the main analysis, a multiple linear regression model adjusting on age, gender, social deprivation (defined by the Epices score), and variables associated in univariate analyses (*p* < 0.20). We did not include the variables that allowed the calculation of the Epices score, the other health outcomes (e.g. the perceived health VAS), nor the five dimensions of the CRA questionnaire (because it was recorded in a subsample of the study population only). The linearity between FI-CD and quantitative variables was assessed using cubic spline functions. Results are presented as beta [95% confidence interval (CI)]. Interactions between age and other explanatory variables were tested. Regression diagnostics were performed.

Moreover, we conducted two sensitivity analyses to evaluate the robustness of the results of the main model, in particular the social deprivation. Firstly, we directly adjusted for the values of the Epices score (as a quantitative variable) instead of the social deprivation (as a binary variable); the coefficient (Beta [95% CI] corresponds to an increase in the FI-CD per a 10-point increase of the Epices score (model 2). Secondly, we conducted a logistic binomial regression model using a binary categorization of the FI-CD as the dependent variable: frail (including severe frail) versus not frail, using the same covariables as in the main model; results are presented as Odds ratios (OR) [95% CI] (model 3).

All statistical analyses were performed using R software, version 3.6.2 (R Core Team (2019). R: A language and environment for statistical computing. R Foundation for Statistical Computing, Vienna, Austria). The threshold for statistical significance was set to *p* < 0.05.

## Results

A total of 117 caregivers were included in the study. Among them, 111 were analyzed (six caregivers were excluded because of a rate > 20% of missing data for variables included in the FI-CD).

The 34 variables included in the FI-CD are described in Supplemental Table 1. The mean ± SD of the 34-item FI-CD was 0.22 ± 0.08; 2.5th and 97.5th percentiles were 0.08 and 0.39, respectively. Forty (36%) individuals were classified as frailty and three (2.7%) as severe frailty. Fifty-seven (51.4%) individuals had at least one missing value for variables included in the FI-CD. Compared to them, individuals without missing value had a similar mean FI-CD (0.22 ± 0.07 versus 0.22 ± 0.09, *p* = 0.97).

**Fig. 1:**
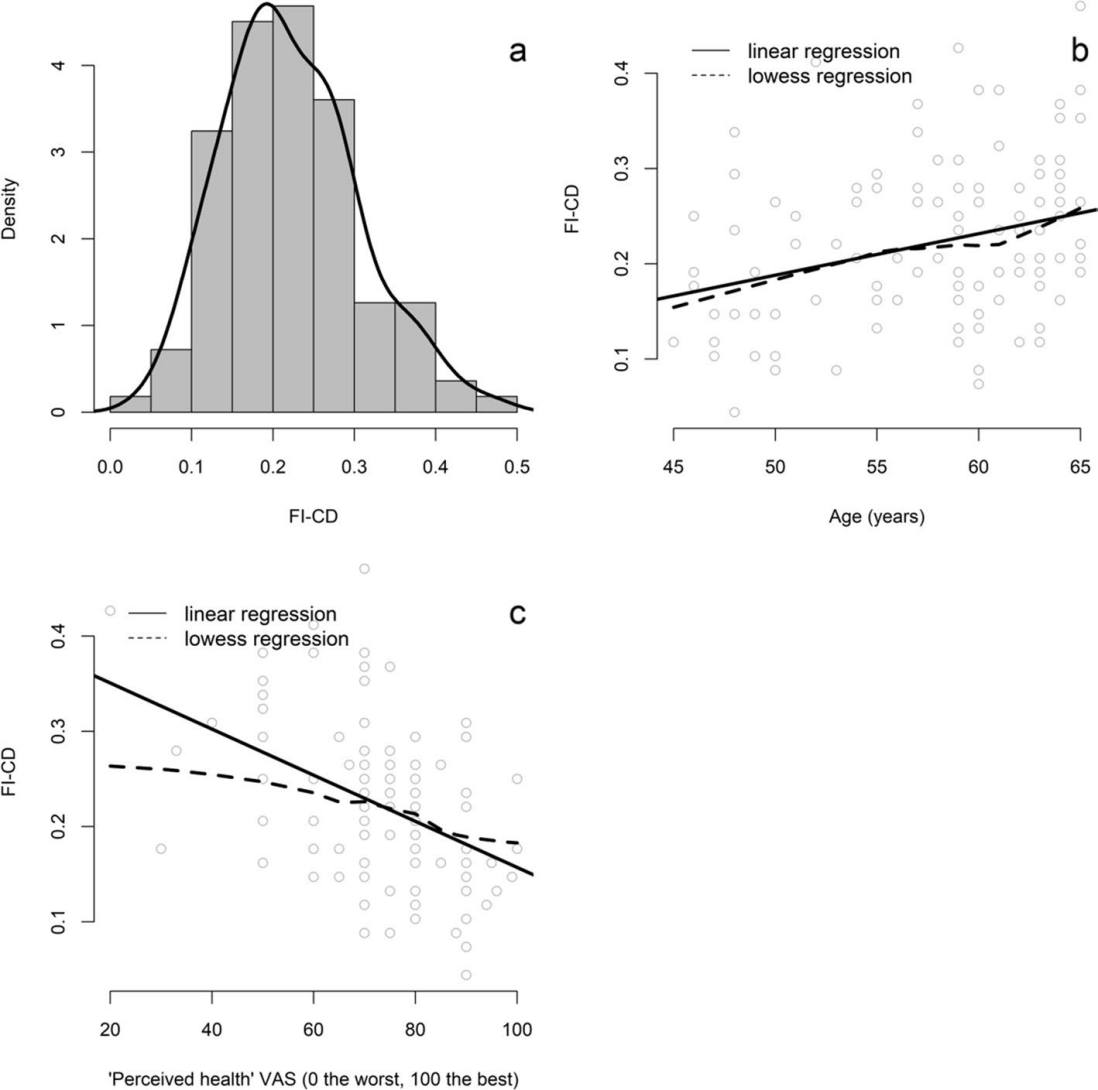
**a** Histogram with the density curve of the frailty index of cumulative deficits (FI-CD). **b** Plot of FI-CD vs. age; the solid line represents the linear regression and the dotted line represents the lowess regression. **c** Plot of FI-CD vs. perceived health visual analog scale (VAS); the solid line represents the linear regression and the dotted line represents the lowess regression

The distribution of the FI-CD was slightly skewed (Fig. 1 (a)). We observed a linear relationship between FI-CD and age (beta [95% CI] = 0.004 [0.002; 0.007] per 1-year increase) (Fig. 1 (b)). The average rate of accumulation of deficits was 2.3% per year of age. We observed an almost linear relationship between FI-CD and perceived health VAS (beta [95% CI] = − 0.024 [− 0.034; − 0.014] per 10-point increase) (Fig. 1 (c)). The FI-CD was also associated with financial assistance for long-term illness (mean FI-CD = 0.21 ± 0.08 versus 0.26 ± 0.09 in individuals without and with long-term illness, respectively, *p* = 0.009).

Characteristics of participants are described in Table 2. Median age [IQR] was 59 [9] years. Most of caregivers were women (78.4%), had tertiary educational level (60.4%), were active (62.2%), in couple (64.6%), and cared for their parent (71.3%). Among the 90 caregivers for which the Epices score was calculable, 20 (22.2%) individuals had a social deprivation. Compared to them, non-precarious caregivers had a higher age (60 versus 55 years, *p* = 0.021), a lower mean FI-CD (0.21 ± 0.07 versus 0.26 ± 0.11, *p* = 0.015) and a higher perceived health VAS (75/100 versus 60/100, *p* = 0.001). We observed a statistical difference between these two groups for all socioeconomic variables (Table 2).

**Table 2:** Characteristics of population in precarious and non-precarious caregivers (*n* = 111)

Variables	N. of data available	Whole population (n = 111)	Non-precarious caregivers (***n*** = 70) ^**(a)**^	Precarious caregivers (***n*** = 20) ^**(a)**^	p
**Sociodemographic characteristics**					
Age (years)	111	59 [9]	60 [8]	55 [11.5]	0.021
Female gender	111	87 (78.4)	56 (80)	13 (65)	0.23
Educational level:	111				< 10^−3^
Primary		19 (17.1)	8 (11.4)	8 (40)	
Secondary		25 (22.5)	13 (18.6)	7 (35)	
Tertiary		67 (60.4)	49 (70)	5 (25)	
Professional situation:	111				< 10^−3^
Active		69 (62.2)	42 (60)	13 (65)	
Inactive		12 (10.8)	4 (5.7)	7 (35)	
Retired		30 (27)	24 (34.3)	0 (0)	
Socio-professional category (current or past):	110				0.024
High profession		31 (28.2)	22 (31.4)	2 (10)	
Intermediate profession		71 (64.6)	44 (62.9)	15 (75)	
Trader, craftsman or farmer		4 (3.6)	3 (4.3)	0 (0)	
Worker		4 (3.6)	1 (1.4)	3 (15)	
Civil status:					< 10^−3^
Couple	110	71 (64.6)	52 (74.3)	6 (30)	
Single	110	39 (35.5)	18 (25.7)	14 (70)	
Financial difficulties for needs (food, rent or charges)	106	16 (15.1)	4 (5.7)	10 (50)	< 10^−3^
Homeowner	108	88 (81.5)	68 (97.1)	5 (25)	< 10^−9^
Leisure within the year (sport, show or vacation)	110	101 (91.8)	70 (100)	11 (55)	< 10^−6^
Epices score ^(a)^	90	9.8 [25.7]	6.8 [11.8]	48.5 [15.2]	< 10^−9^
**Caregiving characteristics**					
Relationship to care recipient	101				0.174
Parent		72 (71.3)	43 (65.2)	16 (84.2)	
Child		10 (9.9)	9 (13.6)	0 (0)	
Spouse		7 (6.9)	4 (6.1)	2 (10.5)	
Other		12 (11.9)	10 (15.2)	1 (5.3)	
Care recipient with neurological disease	100	59 (59)	33 (50.8)	15 (79)	0.036
CRA questionnaire scores for the five dimensions:					
Caregiver’s self-esteem problems	60	18 [6]	18 [5.5]	14.5 [8]	0.62
Lack of family support	58	13.5 [8]	11.5 [6.8]	17 [7]	0.050
Financial problems	56	7 [5]	6 [2.5]	8.5 [4]	0.06
Disrupted schedule	60	15 [8]	12.5 [7.8]	15 [7.3]	0.09
Health problems	58	10 [4.8]	10 [4.5]	9.5 [6.8]	0.57
**Health characteristics**					
Frailty index	111	0.22 ± 0.08	0.21 ± 0.07	0.26 ± 0.11	0.015
Frailty index in class:	111				0.007
Not frail		68 (61.3)	49 (70)	7 (35)	
Frail		40 (36)	20 (28.6)	11 (55)	
Severely frail		3 (2.7)	1 (1.4)	2 (10)	
Perceived health VAS	110	75 [10]	75 [10]	60 [22.5]	0.001
Financial assistance for long-term illness	100	21 (21)	12 (18.2)	6 (31.6)	0.22
Smoking status:	110				0.005
Never smoker		53 (48.2)	38 (54.3)	5 (25)	
Former smoker		46 (41.8)	28 (40)	9 (45)	
Current smoker		11 (10)	4 (5.7)	6 (30)	
AUDIT questionnaire for alcohol misuse:	105				0.55
No misuse		97 (92.4)	63 (94)	18 (90)	
Harmful drinking		5 (4.8)	3 (4.5)	1 (5)	
Alcohol dependence		3 (2.9)	1 (1.5)	1 (5)	
General practitioner consulted within the year	110	109 (99.1)	69 (98.6)	20 (100)	> 0.99
Mammography screening within two years	81	68 (84)	47 (87)	7 (63.6)	0.08
Cervical smear screening within three years	80	60 (75)	42 (79.3)	7 (58.3)	0.15
Colorectal cancer screening within two years	95	40 (42.1)	25 (41)	10 (58.8)	0.27

*CRA* Caregiver Reaction Assessment, *VAS* Visual Analog Scale, *AUDIT* Alcohol Use Disorders Identification Test

^a^ Epices score was available in 90/111 individuals, allowing to define 70 non-precarious and 20 precarious caregivers. Quantitative variables are described using mean ± standard deviation, or median [interquartile range] in case of non-normality and qualitative variables are described using number (percentage)

Univariate analyses between FI-CD and characteristics of caregivers are presented in Table 3. The FI-CD was significantly associated with age (p = 0.001), Epices score (*p* = 0.011), social deprivation (p = 0.015), financial difficulties for needs (*p* = 0.037), not be homeowner (*p* = 0.039), not had leisure within the year (0.001), perceived health VAS (*p* < 0.001) and financial assistance for long-term illness (*p* = 0.009).

**Table 3:** Univariate analyses between the frailty index and characteristics of caregivers

Variables	N. of data available	Beta [95% CI]	p
**Sociodemographic characteristics**			
Age (per 1-year increase)	111	0.004 [0.002; 0.007]	0.001
Gender (male vs. female gender)	111	− 0.018 [− 0.056; 0.02]	0.36
Educational level:	111		
Primary (reference)		–	–
Secondary		−0.027 [− 0.075; 0.022]	0.28
Tertiary		−0.054 [− 0.095; − 0.012]	0.012
Professional situation:	111		
Active (reference)		–	–
Inactive		0.061 [0.01; 0.111]	0.019
Retired		0.018 [−0.017; 0.054]	0.31
Socio-professional category (current or past):	110		
High profession (reference)		–	–
Intermediate profession		0.002 [−0.034; 0.037]	0.93
Trader, craftsman or farmer		−0.019 [− 0.106; 0.068]	0.67
Worker		0.044 [− 0.043; 0.131]	0.32
Civil status (single vs. couple)	110	0.031 [−0.001; 0.064]	0.06
Financial difficulties for needs	106	0.046 [0.003; 0.089]	0.037
Homeowner	108	−0.042 [− 0.082; − 0.002]	0.039
Leisure within the year	110	−0.096 [− 0.15; − 0.041]	0.001
Epices score (per 10-point increase)	90	0.010 [0.003; 0.018]	0.011
Social deprivation (precarious vs. non-precarious)	90	0.048 [0.01; 0.087]	0.015
**Caregiving characteristics**			
Relationship to care recipient	101		
Parent (reference)		–	–
Child		−0.001 [− 0.054; 0.051]	0.96
Spouse		−0.003 [− 0.065; 0.059]	0.92
Other		−0.038 [− 0.087; 0.011]	0.13
Care recipient with neurological disease	100	0.008 [−0.024; 0.040]	0.61
CRA questionnaire scores for the five dimensions:			
Caregiver’s self-esteem problems (per 1-point increase)	60	0.001 [−0.004; 0.005]	0.80
Lack of family support (per 1-point increase)	58	0.003 [−0.001; 0.007]	0.18
Financial problems (per 1-point increase)	56	0.005 [−0.001; 0.011]	0.08
Disrupted schedule (per 1-point increase)	60	0.003 [−0.001; 0.006]	0.11
Health problems (per 1-point increase)	58	0.004 [−0.001; 0.009]	0.15
**Health characteristics**			
Perceived health’ VAS (per 10-point increase)	110	−0.024 [− 0.034; − 0.014]	< 10^−3^
Financial assistance for long-term illness	100	0.052 [0.013; 0.09]	0.009
Smoking status:	110		
Never smoker (reference)		–	–
Former smoker		−0.002 [−0.035; 0.031]	0.91
Current smoker		−0.018 [− 0.072; 0.036]	0.51
Audit questionnaire for alcohol misuse:	105		
No misuse		–	–
Harmful drinking		0.012 [−0.064; 0.088]	0.76
Alcohol dependence		−0.052 [− 0.149; 0.046]	0.29
General practitioner consulted within the year	110	0.061 [−0.104; 0.227]	0.47
Mammography screening within two years	81	−0.02 [− 0.068; 0.029]	0.42
Cervical smear screening within three years	80	−0.001 [− 0.043; 0.041]	0.97
Colorectal cancer screening within two years	95	−0.004 [− 0.038; 0.031]	0.84

*95% CI* 95% Confidence Interval, *CRA* Caregiver Reaction Assessment, *VAS* Visual Analog Scale, *Audit* Alcohol Use Disorders Identification Test

Multivariate analyses between FI-CD and characteristics of caregivers are presented in Table 4. The FI-CD was significantly associated with age (beta [95% CI] = 0.005 [0.002; 0.009] per 1-year increase, *p* = 0.005) and social deprivation (beta [95% CI] = 0.054 [0.007; 0.102], *p* = 0.025).

**Table 4:** Multivariate analyses between the frailty index and characteristics of caregivers (*n* = 90)

Variables	Main model		Model 2		Model 3	
	Beta [95% CI]	p	Beta [95% CI]	p	OR [95% CI]	p
**Sociodemographic characteristics**						
Age (per 1-year increase)	0.005 [0.002; 0.009]	0.005	0.005 [0.002; 0.008]	0.005	1.08 [0.97; 1.21]	0.16
Gender (male vs. female gender)	−0.012 [− 0.051; 0.027]	0.56	−0.004 [− 0.042; 0.034]	0.84	0.57 [0.15; 1.85]	0.36
Educational level:						
Primary (reference)	–	–	–	–	–	–
Secondary	−0.011 [− 0.062; 0.04]	0.67	− 0.012 [− 0.063; 0.038]	0.63	0.28 [0.05; 1.36]	0.12
Tertiary	−0.012 [− 0.058; 0.034]	0.61	− 0.011 [− 0.058; 0.035]	0.62	0.49 [0.13; 1.94]	0.30
Professional situation:						
Active (reference)	–	–	–	–	–	–
Inactive	0.008 [−0.047; 0.063]	0.77	0.013 [−0.041; 0.067]	0.63	5.37 [0.96; 43.7]	0.07
Retired	−0.024 [− 0.069; 0.021]	0.29	− 0.024 [− 0.069; 0.021]	0.29	1.08 [0.29; 4.03]	0.91
Epices score (per 10-point increase)	–	–	0.011 [0.002; 0.020]	0.020	–	–
Social deprivation (precarious vs. non-precarious)	0.054 [0.007; 0.102]	0.025	–	–	5.11 [1.23; 23.8]	0.028

The main model and model 2 are multiple linear regressions with the frailty index (as a quantitative variable) as the dependent variable. The model 3 is a binomial logistic regression with the frailty status (frail vs. not frail) as the dependent variable

*95% CI* 95% Confidence Interval, *OR* Odds Ratio

**Fig. 2:**
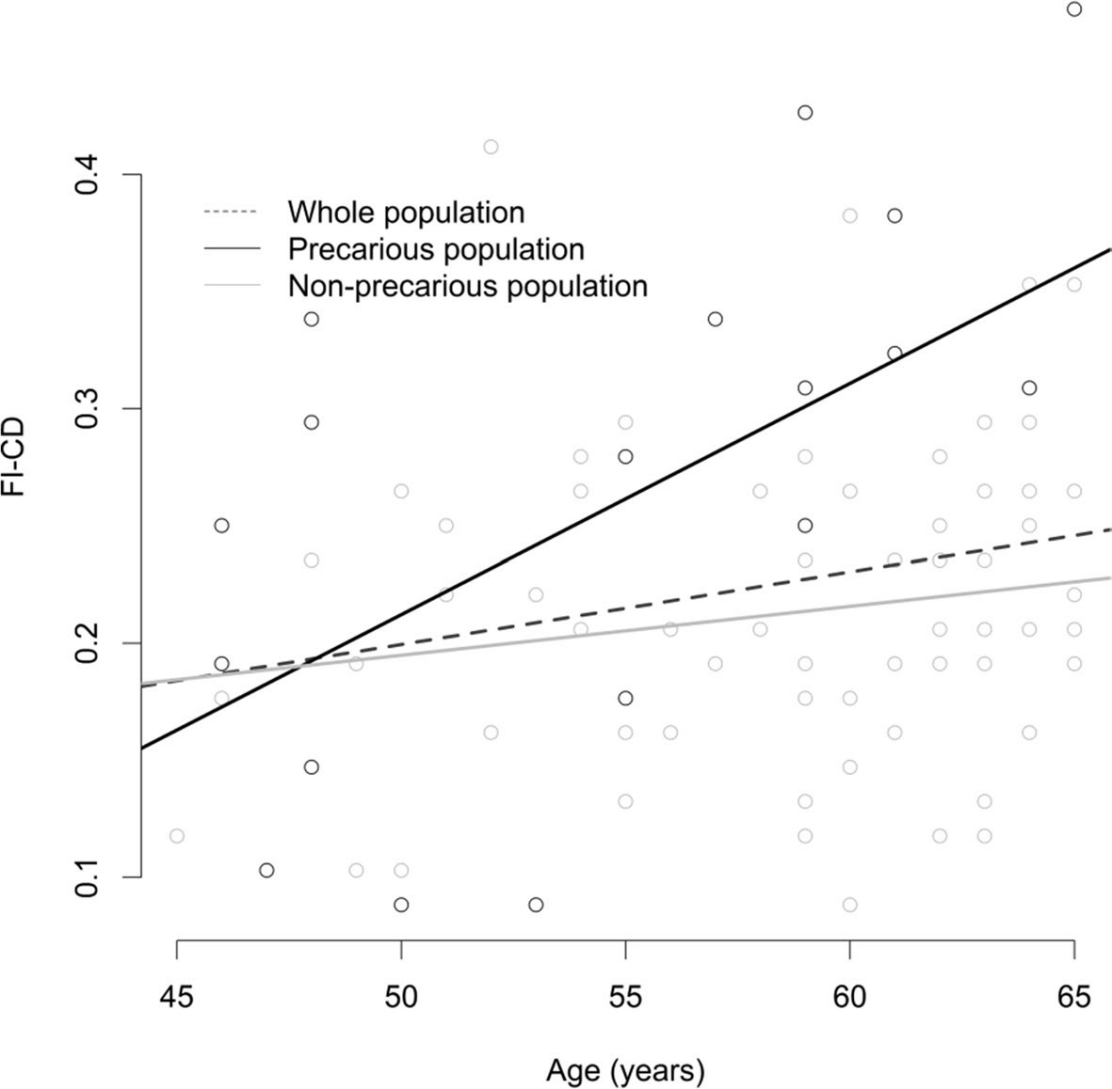
Plot of the frailty index of cumulative deficits (FI-CD) vs. age; in the whole population (in blue), non-precarious (in green) and precarious (in red) caregivers

Interactions between age and other explanatory variables were tested in the main model. Firstly, a significant interaction was observed between age and gender (p = 0.005). The adjusted relationship between FI-CD and age was: beta [95% CI] = 0.016 [0.008; 0.024], *p* = 0.001 in men, and beta [95% CI] = 0.003 [− 0.001; 0.007], *p* = 0.10 in women. Secondly, a significant interaction was observed between and age and social deprivation (*p* = 0.036). The adjusted relationship between FI-CD and age was: beta [95% CI] = 0.010 [0.002; 0.019], *p* = 0.017 in precarious caregivers, and beta [95% CI] = 0.003 [− 0.001; 0.007], *p* = 0.19 in non-precarious caregivers. The interaction between age and social deprivation is illustrated in Fig. 2.

We conducted two sensitivity analyses. Model 2 showed a significant association between FI-CD and age (beta [95% CI] = 0.005 [0.002; 0.008] per 1-year increase, *p* = 0.005) and Epices score (beta [95% CI] = 0.011 [0.002; 0.020] per 10-point increase, *p* = 0.020). Model 3 showed a significant association between frailty and social deprivation (OR [95% CI] = 5.11 [1.23; 23.8], *p* = 0.028).

## Discussion

A 34-item frailty index of cumulative deficits was constructed using data from a large, multidisciplinary preventive health check in French middle-aged caregivers, and used to study the associated factors, in particular socioeconomic conditions. In multivariate analysis, the FI-CD was associated with age and social deprivation, measured using the Epices score.

### Methodological issues

Although some tools are widely used, as the clinical frailty score described by Fried et al. [1], there is no gold standard for frailty assessment that could be used as a validation method. Predictive validation against mortality is a robust method of validation [12]; however, data on mortality or other longitudinal outcomes were not available in our cross-sectional study, which was the main limit of this work. However, we constructed this FI-CD with a sufficient number of deficits (> 30), as recommended [12]. Indeed, although the approach is relatively simple, the results yielded by the FI-CD have been consistent between studies even though not every FI-CD considers the same deficits, or even the same number of deficits; and the frailty index is strongly associated with the risk of death, institutionalization and worsening health status, especially when at least 30 variables are included [12]. FI-CD used in numerous studies are well validated [9], and have been applied to multiple datasets, mainly in older people [11, 12, 28, 35–39], but also in younger individuals [13, 40–46], as well as in animal models [47, 48], that can give assurance of the robustness of the approach [12]. The rate of accumulation of deficits was 2.3% per year, which was similar to other studies (e.g. baseline and follow up rate was 2.0 and 2.6% per year, respectively, in the study of Searle, et al. [12], 3% in the study of Rockwood and Mitnitski [11]). In addition, the FI-CD was associated with other health outcomes (perceived health VAS and financial assistance for long-term illness). Therefore, it seems valid to use this 34-item FI-CD for clinical practice in the population of middle-aged caregivers.

For the construction of the FI-CD, several quantitative variables were categorized, but not all these had validated cut-points (e.g. the measure of sarcopenia using dual energy X-ray absorptiometry), which is a frequent limit of studies building a FI-CD. Another limitation was that the study population consisted of voluntary caregivers, not from a representative sample. Compared with the population of the north of France, we have observed an over-representation of women and individuals with a higher educational level and higher profession, which is usual with this type of recruitment. Selection bias limits the generalization of the results; however, they do not invalidate the construction of the FI-CD or the study of associated factors. Furthermore, missing values were observed, especially for the CRA questionnaire that was implemented during the study, which prevented from integrating it in multivariate models. Finally, some useful data were not available, e.g. the number of hours spent on caregiving.

Fried’s frailty approach, altought very popular in studies on the loss of autonomy, seemed less suitable in studies on aging well. We demonstrate in this study that the FI-CD is easily implemented and well suited to this purpose. The 34-item FI-CD allowed to identify 40 (36%) individuals with frailty and 3 (2.7%) with severe frailty. As frailty is a decline in functioning across multiple physiological systems, such a tool increases clinical judgement in the management of the population of middle-aged caregivers. It will be useful in selecting caregivers, to whom to offer coaching, the second step of the preventive program of the Institut Pasteur of Lille which aims to correct frailty in this population.

The 34-item FI-CD also allowed to study factors associated with frailty in middle-aged caregivers. We focused on socioeconomic factors, in order to identify sub-populations at risk and guide the future recruitment of caregivers. We observed univariate associations between FI-CD and financial difficulties for needs, not being homeowner, and not having leisure within the year, which were components of the Epices score. We also observed non-significant associations with several dimensions of the CRA questionnaire (in particular financial problems), probably due to a lack of statistical power, because these data were available only in a sub-sample of the study population. Social deprivation was associated with FI-CD in all multivariate models: in the main model (beta [95% CI] = 0.054 [0.007; 0.102], *p* = 0.025 for precarious), in the model using the Epices score as a quantitative variable (beta [95% CI] = 0.011 [0.002; 0.020] per 10-point increase, *p* = 0.020), as well as in the logistic model (OR [95% CI] = 5.11 [1.23; 23.8], *p* = 0.028 for precarious). These results are consistent with previous studies which showed associations between low socioeconomic condition and frailty [44–46, 49–52]. More specifically, it is known that caregivers in social deprivation are at higher risk of caregiver burden. Indeed, previous studies revealed that risk factors for caregiver burden include low educational attainment, social isolation and financial stress [53]. For these reasons, social support is a key target of interventions to reduce caregiver burden. This result is all the more interesting since there are a certain number of social or financial aids in France, which are not necessarily requested by individuals who can subscribe to them, due to lack of knowledge of the existence of these aids or because of administrative difficulties (especially in people with low level of education). Integrating social support into a health prevention program could therefore be an important lever for improving the health of precarious caregivers. Furthermore, we observed a significant interaction between age and social deprivation. Then, beyond the association between FI-CD and social deprivation, this result suggests that the difference in the level of frailty between precarious and non-precarious increases over time, which highlights the value of an early care in this population at risk.

## Conclusions

The results of this study suggested that the 34-item frailty index built using data from the large, multidisciplinary health check of the Institut Pasteur of Lille could have clinical utility, augmenting clinical judgement in the management of middle-aged caregivers. Social deprivation appeared as an important factor associated with frailty in this population, highlighting the importance of early care and social support for precarious caregivers, and guiding the future recruitment of the preventive program.

## Supplementary Information

**Additional file 1: Supplemental Table 1**. Description of variables included in the Frailty Index.

## Data Availability

The datasets generated and/or analyzed during the current study are not publicly available due to legal and ethical reasons (no consent from participants, impossibility of anonymizing and maintaining statistical confidentiality due to the large number of variables and the relative low number of participants) but are available from the corresponding author on reasonable request.

## Abbreviations

FI-CD: Frailty index of cumulative deficit
BMI: Body mass index
CRA: Caregiver Reaction Assessment
VAS: Visual Analog Scale
Audit: Alcohol Use Disorders Identification Test
Lowess: Locally weighted scatterplot smoothing
SD: Standard deviation
IQR: Interquartile range
CI: Confidence interval
OR: Odds ratio
