# Supplementary material for: The relationship between social deprivation and a frailty index of cumulative deficits in French middle-aged caregivers
Source: BMC Geriatr. 2022 Jan 3;22:15. doi: 10.1186/s12877-021-02736-3 (PMC8721985; doi:10.1186/s12877-021-02736-3)
Supplement: Supplementary file 1 — Additional file 1: Supplemental Table 1. Description of variables included in the Frailty Index. [file 12877_2021_2736_MOESM1_ESM.docx]

**Supplemental Table 1. Description of variables included in the Frailty Index.**

| **Variables** | **N. of data available** | **n (%)** |
| --- | --- | --- |
| **Cognition and autonomy** |  |  |
| Cognitive assessment (MoCA score), Fragility | 108 | 9 (8.3) |
| Processing speed assessment (Coding score), Fragility | 107 | 6 (5.6) |
| Autonomy assessment (4-item IADL score), Fragility | 108 | 9 (8.3) |
| Able to manage his/her daily life if alone for 15 days, No | 109 | 6 (5.5) |
| **Dietetics** |  |  |
| Overall nutritional intakes, Insufficient | 109 | 4 (3.7) |
| Calcium intakes, Insufficient | 110 | 49 (44.6) |
| Protein intakes, Insufficient | 111 | 21 (18.9) |
| **Physical activity and risk of falling** |  |  |
| Marshall physical activity assessment score, Insufficient | 110 | 71 (64.6) |
| One or more falls in the past year, Yes | 108 | 19 (17.6) |
| Gait speed test (4-metre), Fragility | 110 | 3 (2.7) |
| Handgrip strength test, Fragility | 110 | 6 (5.5) |
| **Comorbidities** |  |  |
| Cardiovascular disease, Yes | 108 | 2 (1.9) |
| Other heart disease, Yes | 108 | 7 (6.5) |
| High blood pressure, Yes | 107 | 42 (39.3) |
| Diabetes, Yes | 106 | 2 (1.9) |
| Dyslipidaemia, Yes | 108 | 80 (74.1) |
| Cancer, Yes | 110 | 10 (9.1) |
| Airway obstruction, Yes | 110 | 9 (8.2) |
| Thyroid disease, Yes | 104 | 8 (7.7) |
| Vision disease, Yes | 106 | 13 (12.3) |
| Anxiety: | 104 |  |
| Yes |  | 6 (5.8) |
| Doubt |  | 21 (20.2) |
| No |  | 77 (74) |
| Depression: | 101 |  |
| Yes |  | 30 (29.7) |
| Doubt |  | 27 (26.7) |
| No |  | 44 (43.6) |
| **Regular treatment** | 108 | 71 (65.7) |
| **Functional signs** |  |  |
| Chronic sleep disorders, Yes | 103 | 64 (62.1) |
| Difficulty retaining urine, Yes | 111 | 25 (22.5) |
| **Lab values and paraclinical examinations** |  |  |
| Anaemia | 108 | 5 (4.6) |
| Elevated hs-CRP | 108 | 23 (21.3) |
| Elevated liver enzymes (ALAT, ASAT or GGT) | 108 | 12 (11.1) |
| Body mass index: | 109 |  |
| Obesity |  | 24 (22) |
| Overweight |  | 27 (24.8) |
| Normal |  | 54 (49.5) |
| Leanness |  | 4 (3.7) |
| Pure tone audiometry, Deficit |  | 16 (14.8) |
| Hip bone mineral density: | 111 |  |
| Osteoporosis |  | 7 (6.3) |
| Osteopenia |  | 50 (45.1) |
| Normal |  | 54 (48.7) |
| Lumbar spine bone mineral density: | 111 |  |
| Osteoporosis |  | 19 (17.1) |
| Osteopenia |  | 36 (32.4) |
| Normal |  | 56 (50.5) |
| Dual energy X-ray absorptiometry, Sarcopenia | 111 | 49 (44.1) |
| Electrocardiogram, Anomalies | 101 | 25 (24.8) |

MoCA: Montreal Cognitive Assessment; IADL: Instrumental Activities of Daily Living; hs-CRP: high-sensitivity C-Reactive Protein; ASAT: Aspartame Aminotransferase; ALAT: Alanine Aminotransferase; GGT: Gamma-GT.
